# Emerging Patient-Driven Health Care Models: An Examination of Health Social Networks, Consumer Personalized Medicine and Quantified Self-Tracking

**DOI:** 10.3390/ijerph6020492

**Published:** 2009-02-05

**Authors:** Melanie Swan

**Affiliations:** Research Associate, MS Futures Group, P.O. Box 61258, Palo Alto, CA 94306, USA

**Keywords:** Patient-driven health care, health social networks, personalized medicine, quantified self-tracking, health care delivery, predictive health care, preventive health care, long-tail medicine, Internet, open source

## Abstract

A new class of patient-driven health care services is emerging to supplement and extend traditional health care delivery models and empower patient self-care. Patient-driven health care can be characterized as having an increased level of information flow, transparency, customization, collaboration and patient choice and responsibility-taking, as well as quantitative, predictive and preventive aspects. The potential exists to both improve traditional health care systems and expand the concept of health care though new services. This paper examines three categories of novel health services: health social networks, consumer personalized medicine and quantified self-tracking.

## 1. Introduction

The life sciences field is advancing and changing in nearly every dimension, both content-wise and structurally. Volumes of new content are coming forth in the form of key research findings, affordable new technologies and simultaneous holistic and reductionist expansions via systems biology approaches and new sub-field branching. Structurally, life sciences are changing in three important ways: the concept of life sciences, how science in general is conducted and the models by which health and health care are understood and realized.

Conceptually over time, life sciences are transitioning from being an art to a science to an information technology to now, an engineering problem. The ways of science are also changing, both in how it is being conducted and in who is conducting it. The historical notion of science consisted of investigating and enumerating physical phenomena and doing hypothesis-driven trial and error experimentation. An evolving notion of science adds three additional steps to the traditional method to create a virtuous feedback loop: first, mathematical modeling as a means of more actively understanding phenomena and predicting outcomes [1], second, software simulation for conducting exponentially many experiments, possibly with the freedom to analyze broader emergences outside of the constraints of hypothesis-formation [2], and third, in some cases, demonstrating mastery of the phenomena by building real lab samples using synthetic biology and other techniques [3]. Who is conducting science is changing as the notion of being in a post-scientific society explains; innovation is occurring in more venues, not just governmental and industrial research labs but increasingly at technology companies, startups, small-team academic labs and by creative entrepreneurs and other individuals [4].

**Figure 1.**
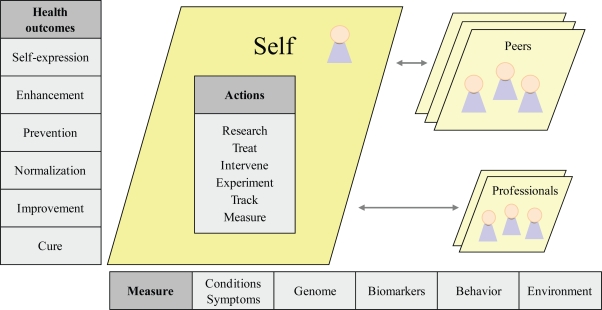
A new model of health and health care.

How health and health care are understood and realized is the third important way that the structure of life sciences is changing. Webster’s dictionary defines health care as the “efforts made to maintain or restore health especially by trained and licensed professionals” (http://www.merriam-webster.com/dictionary/healthcare). Figure 1 takes this as a base and illustrates a new model of health and health care. The left column depicts the expanded definition of health and the continuum of health outcomes. The initial focus on illness cure is broadened to include the improvement or resolution of chronic ailments, the attainment of baseline health normalization, the prevention of unhealthy states and promotion of wellness, the enhancement of current genetic, physical and mental health and the notion of health possibly being a vehicle for creative self-expression.

Along the bottom are the aspects of health to measure, starting with the usual conditions and symptoms and now including genomic testing, a wider range of ongoing blood-based and other biomarker testing, behavioral tracking such as nutritional intake, exercise and sleep and more systemically, evaluating and monitoring a person’s environment.

The body of Figure 1 shows the participants of the multi-party health care system and their relative importance from the reference frame of the individual. Individuals themselves can be the focus and center of action-taking in a number of areas such as measuring, tracking, experimenting and engaging in interventions, treatments and research. Consumers are starting to do this individually, in collaboration with health peers, who also have greater prominence now, and in co-care with physicians and other medical professionals. Box 1 provides a look at how some of the industry’s key thinkers are characterizing the changing definition of health and health care.

**Box 1. Quoted excerpts regarding the changing definition of health and heath care.**

“…the NHS [National Health Service] of the future will be one of patient power, patients engaged and taking control over their own health and healthcare.”

– Gordon Brown, U.K. Prime Minister (http://socialhealthnetwork.org)

“The premise is that we are at a new phase of health and medical care, where more decisions are being made by individuals on their own behalf, rather than by physicians, and that, furthermore, these decisions are being informed by new tools based on statistics, data, and predictions … We will act on the basis of risk factors and predictive scores, rather than on conventional wisdom and doctors recommendations. We will act in collaboration with others, drawing on collective experience with health and disease … these tools will create a new opportunity and a new responsibility for people to act - to make health decisions well before they become patients.”

– Thomas Goetz, Decision Tree (http://thedecisiontree.com/blog/?p=278)

“We believe that the new generation of web services will change the way medicine is practiced and healthcare is delivered.”

– Bertalan Meskó, medical blogger (http://scienceroll.com/medicine-20)

### 1.1. Scope of Analysis

This paper focuses primarily on one aspect of the broader shifts in life sciences, how health and health care are understood and realized, particularly through emerging patient-driven health care models. The paper is intended to provide an early view into these models which could potentially have a large future impact but are only just in their beginning phases. The paper does not balance the discussion of the new models with adequate acknowledgement of the contribution of medical professionals working in traditional health care systems. Also in this early survey of the field, the paper only gives anecdotal rather than comprehensive coverage to the many shortcomings of emerging patient-driven health care models. Some of these shortcomings may include potential bias, error, lack of rigor in data collection and analysis, lack of professionalism, possible damage to the self and others, and legal and ethical dimensions. All of these areas could be reviewed more fully in a subsequent and more extensive analysis of the emergence and efficacy of patient-driven health care models as the industry continues to develop.

## 2. Emerging Patient-Driven Health Care Models

Three emerging patient-driven health care models are now discussed in detail: health social networks, consumer personalized medicine and quantified self-tracking.

### 2.1. Health Social Networks

#### 2.1.1. Health social networks introduction

Social networks have become a powerful tool for bringing people with shared interests together to interact. In addition to general social networks (examples: FaceBook, MySpace) and career social networks (examples: LinkedIn, Plaxo), more specific purpose-driven social networks are emerging. In the Finance 2.0 area, social networking has become an overlay or a property of asset and expense management websites like Wesabe, Mint, Zecco, Cake Financial and Expensr. In the health space, over twenty health social networks have launched in the last few years including PatientsLikeMe, CureTogether, DailyStrength, MedHelp, HealthChapter, MDJunction, Experience Project, peoplejam, and OrganizedWisdom (Table 1 has URLs for all health social networks mentioned).

#### 2.1.2. Health social networks definition

**Figure 2.**
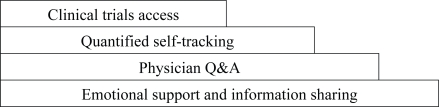
Services provided by health social networks.

A health social network is a website where consumers may be able to find health resources at a number of different levels (Figure 2). Services may range from a basic tier of emotional support and information sharing to Q&A with physicians to quantified self-tracking to clinical trials access.

One key value health social networks provide is the potential to find others in similar health situations and share information about conditions, symptoms and treatments. A health condition is a particularly strong affinity and the collective learning and experience of others can be leveraged and shared to help individuals make decisions. Health social networks are primarily directed at patients but caretakers, researchers and other interested and knowledgeable parties may be able to participate.

The largest and best-known health social network is PatientsLikeMe, which started in 2004 and had, as of December 2008, 26,059 patients (http://www.patientslikeme.com/all/patients). Also as of December 2008, membership was growing 10% per month with the company having the goal of reaching one million patients encompassing 200 different diseases by 2012 [5]. 5% of all amyotrophic lateral sclerosis (ALS) patients in the U.S. are site members; this is the largest current existing data set on the disease [6].

To date, health social networks have been focused mainly on medical conditions for which cures are sought, although some websites have user communities for healthy living. Some health social networks serve as a point resource for over 700 conditions (examples: MDJunction, HealthChapter) and in fact, a key benefit of health social networks is that they can offer a more comprehensive look at a patient’s health by covering a deeper and broader range of conditions than is expedient for traditional medicine. Other health social networks focus on fewer conditions more profoundly (examples: PatientsLikeMe, CureTogether), using additional functionality such as quantified self-tracking and collaborative filtering to identify potentially related conditions patients might be experiencing and match patients in similar situations. Collaborative filtering has been identified as a critical mechanism in facilitating patient information-seeking and trust-building in Internet health models [7].

#### 2.1.3. Services provided by health social networks

This section has an in-depth review of the services provided by health social networks: emotional support and information sharing, physician Q&A, quantified self-tracking and clinical trials access.

##### Emotional support and information sharing

The basic services offered by the majority of health social networks are a mix of emotional support and information sharing at no cost to registered site users. Some health social networks may emphasize one area more, such as information and research citations (example: OrganizedWisdom) or social connection and support (example: DailyStrength). Websites may auto-populate general condition information from Internet health resources such as Wikipedia articles and PubMed links. In addition to the general information, patients may be able to enter qualitative and quantitative data about their own conditions, symptoms, treatments and overall experiences.

**Figure 3.**
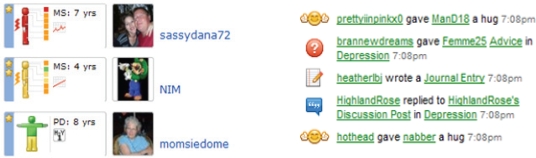
PatientsLikeMe detailed profile examples for an HIV, MS, Parkinson’s Disease and Anxiety patient (left) and DailyStrength activity feed items (right).

Emotional support, social support and patient empowerment are important components of health social networks, available both implicitly and explicitly. Implicitly, emotional support is experienced by seeing that there are others with similar conditions, that “I am not alone.” Implicit emotional support is also felt by being a community member, participating in the process of creating a personal profile (Figure 3) and recording health information, seeing how other non-medical professionals describe the same conditions and symptoms and finding out what remedies others have tried. Emotional support is also offered explicitly in some health social networks through user interaction. Site members may have the ability to comment on forums, publicly or privately message each other, give each other advice and transmit lightweight social greetings, such as hugs, as shown in excerpts from DailyStrength’s activity feed (Figure 3).

The impact of emotional support and patient information sharing is thought to be quite positive but is not fully understood yet. PatientsLikeMe has conducted some research, finding that “patients who choose to explicitly share health data within a community may benefit from the process, helping patients engage in dialogues that may inform disease self-management [8].”

##### Physician Q&A

**Figure 4.**
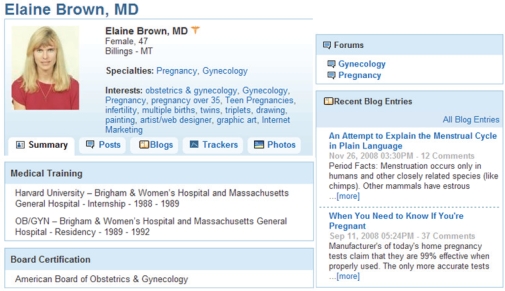
Example of a MedHelp physician homepage.

A second service offered by several health social networks (examples: MedHelp, WellSphere, MDJunction, ehealth forum, iMedix, WeGoHealth) is the ability to pose questions to physicians. Questions and responses are usually displayed publicly unless the patient marks them as private. Posing questions may be free or fee-based, for example at MedHelp, it is $22 to pose a question to a physician directly and free to post a question in the medical communities where peers or professionals may respond. The websites generally have doctor profile pages where physicians complete information about their expertise, background and affiliations, with links to previous question responses on the site and possibly their medical blog entries (Figure 4).

This transparency and willingness to interact helps to start changing the image of doctors as 10-minute diagnosticians to accessible collaborators in care. Many doctors are willing to answer questions and recommend next steps, and possibly provide a preliminary and well-caveated diagnosis. Even this basic mechanism of lightweight doctor-patient interaction could help ease burdens on the health care system. The conventional wisdom may have been that physicians would not take part for legal, reputational and other reasons but the key point is that they are willing to participate and in fact may find reputational enhancement and other benefits.

##### Quantified self-tracking

A third type of service offered by some health social networks (examples: PatientsLikeMe, CureTogether, MedHelp, SugarStats) is quantified self-tracking. The self-tracking functionality consists of easy-to-use data entry screens for condition, symptom, treatment and other biological information. The information can then be seen in a graphical display, possibly with views by individual, aggregated population or custom groups.

**Figure 5.**
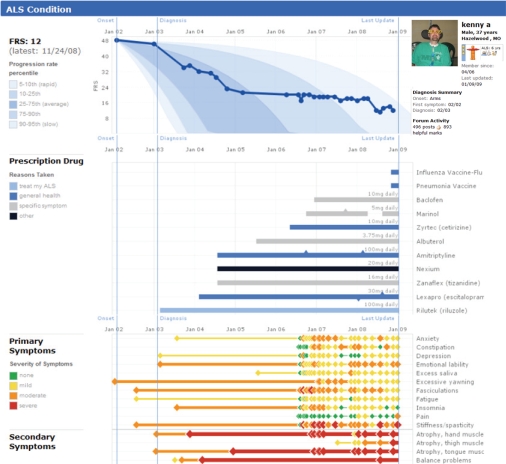
PatientsLikeMe ALS profile charts example.

**Figure 6.**
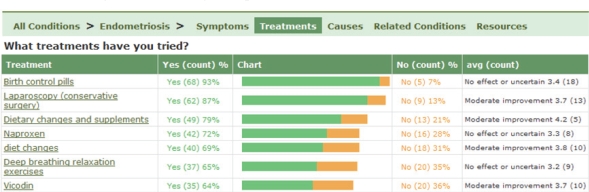
CureTogether patient-entered treatment statistics.

For example, Figure 5 shows a detailed patient profile from PatientsLikeMe including disease progression, prescription drugs and symptom tracking for a 37 year-old male who has had ALS for six years, and Figure 6 shows an aggregated view of the top treatments tried by the CureTogether endometriosis community. Individual tracking data, medications and other relevant information can be printed from the websites to expedite interaction at in-person doctor visits.

**Figure 7.**
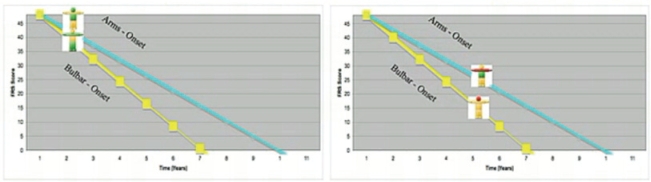
PatientsLikeMe peer disease tracking over time with easy-to-read graphical images of patient status.

Self-tracking information is further incorporated into the PatientsLikeMe site by mapping the data to a graphical representation of the patient as shown in Figure 7, a stick figure shaded with different colors per symptom severity and disease stage so anyone looking at the profile can assess the patient’s status immediately. Figure 7 depicts two patients with ALS, one with arms onset (top diagonal line) and one with bulbar onset (bottom diagonal line), tracking their condition progression by year (x-axis, years 0–10) and decline in Functional Rating Scale (FRS) (y-axis, 0–45). The site’s collaborative filtering allows users to find “patients like me,” which is important since similar others are the most relevant for providing and sharing information.

##### Clinical trials access

A fourth type of service offered by some health social networks is information regarding clinical trials. Even the presence of health social networks makes traditional clinical trials more efficient through the availability of large searchable online databases of patients with health history and condition information. Pharmaceutical companies, industry analysts, policy architects and other interested parties can assess demand and market size directly from health social network websites.

PatientsLikeMe and Inspire are at least two health social networks offering access to clinical trials at present, selling anonymized data to pharmaceutical companies, universities and research labs. For example, in May 2008, Novartis recruited clinical trial participants from PatientsLikeMe estimating that they were able to speed up their 1,200-patient study of a new medicine for multiple sclerosis by a few months [9]. In another instance, PatientsLikeMe contacted 1,500 ALS patients for another research project and received 50 DNA samples (3.3%) [10]. The yield might not seem high but the time and cost savings in identifying, screening, contacting and obtaining responses from relevant patients is significant.

In addition to lower-cost patient recruitment, there are three other ways that health social networks are improving the quality of clinical trials. First, the depth of information generated through large online patient communities creatively interacting and monitoring their conditions with quantitative tracking tools can lead to new findings that give a better understanding of the underlying conditions. PatientsLikeMe in-house research staff is publishing some of these findings, such as the identification of non-motor symptoms of Parkinson’s disease in younger sufferers [11]. Second, health social networks provide a feedback loop to the clinical trials process. For example, PatientsLikeMe patients noticed and suggested corrections and improvements to the graphical display of data in ALS clinical trials [12]. Third, online health tracking in conjunction with clinical trials means that patients can make their experience feedback, including response to drugs, available as a public resource.

The obvious next phase of active patient participation in health social networks is patient-inspired research and patient-run research. This concept is also called open source health research and crowd-sourced health research. Self-run clinical trials and structured self-experimentation is emerging as patients may no longer have the inclination to wait for formal research findings and pharmaceutical company-sponsored clinical trials, and can possibly fill the medicine gap for orphan diseases and other conditions that do not make good business cases in the existing pharmaceutical model. Patients can review research literature and other remedy suggestions on their own and try them, tracking the results in a rigorous manner, sharing the information and running non-traditional clinical trials themselves. In one example, a PatientsLikeMe patient, newly diagnosed with rapidly progressive and young-onset ALS gathered 250 patients to self-experiment with lithium [13] per a research paper he had found [14]. The self-run patient study results were preliminary and found that the use of lithium did not slow disease progression. The example highlights many elements of the new power and role of patients, their ownership of the health care process and the attendant contentious legal, ethical, methodological and other issues.

Inevitably, fraud is likely to arise or may already exist in health social networks as there are significant economic incentives for drugs and other treatments to have high patient usage statistics and favorable reputations. The bona fide peer community may be one of the most helpful resources in detecting and policing fraud due to the deep knowledge of patients regarding their conditions and remedies, and their time spent on the websites.

#### 2.1.4. List of health social networks

A list of current health social networks is presented in Table 1, organized into three categories: patient-focused general multi-condition websites, patient-focused cause-specific websites and physician-focused social networks. Most patient-focused health social networks offer the basic level of service, emotional support and information sharing, for a variety of medical conditions. About half also offer the second level of service, some sort of Q&A with physicians, and a few offer the third and fourth levels of service, quantitative self-tracking and clinical trials access.

A second class of patient-focused health social networks is cause-specific, offering primarily the basic emotional support and information sharing service. Physician-focused health social networks are also starting to have a presence, both for the usual industry-related networking but also as collaboration platforms, notably the Medical Image and Video Exchange (http://www.medting.com) which in December 2008 had over 2,011 cases with 17,812 images and videos uploaded and available for collaboration, and OR-Live, which presents live interactive webcasts of surgical procedures to physicians, patients and the public.

### 2.2. Consumer Personalized Medicine

#### 2.2.1. Consumer personalized medicine introduction

In the last several decades, advances in science have been enabling new paradigm understandings of biological life. Molecular biology was one such key shift, genomics is another that is occurring now, and proteomics, metabolomics and any or all of the other twenty “omics” fields (http://omics.org) may further revolutionize the understanding and management of all biological processes. Current unsolved disease states such as cancer are complex and expressed differently in diverse groups of patients. Initially, it is easiest to tackle cases where the patients have certain characteristics or the disease is expressed in certain ways. Therefore therapies are targeted to sub-groups of patients, for example Imatinib for certain chronic myelogenous leukemia (CML) patients and Tamoxifen for certain breast cancer patients.

It is starting to be realized that obtaining and understanding a whole new level of detailed individual biological information could be necessary for advances in both institutional medicine and patient-driven medicine. The required information is starting to be known to the consumer and is increasingly available publicly, without the individual needing to wait for a condition to advance to the stage of symptoms or for a doctor to know that the information is available and relevant or notice that a particular condition could be assessed via testing. Consumers are taking it upon themselves to obtain a better understanding, resolution and possible prevention of disease conditions.

#### 2.2.2. Consumer personalized medicine definition

The core definition of personalized medicine is using an individual’s specific biological characteristics to tailor therapies to that person, including drugs, drug dosage and other remedies. There are other more expansive descriptions, for example, as offered by the Personalized Medicine Coalition (http://www.personalizedmedicinecoalition.org/sciencepolicy/personalmed-101_overview.php). Part of the change in how health and health care are now being understood and realized is in using systemic personalized medicine approaches to individuals (Box 2). A systemic approach may incorporate a combination of an individual’s genetic, blood and other biomarker, environmental, lifestyle and other data. Consumer personalized medicine is the further step of individuals collecting and synthesizing their own data and using it to proactively manage their health.

**Box 2. Quoted excerpts regarding a systemic approach to personalized medicine.**

“The health of each person is a unique combination of genetic, environmental and lifestyle factors. Not everyone who has a disease has it for the same reasons or with the same severity.”

– Alex Bangs, CEO Entelos (http://entelos.com/virtualPatients.php)

“The Genetics and Public Policy Center at Johns Hopkins University finds that four in five Americans support the idea of a nationwide study to investigate the interactions of genes, environment and lifestyle, and three in five would be willing to take part in such a study.”

– David Duncan, ExperimentalMan (http://experimentalman.com/blog/?p=55)

“The National Institute of Standards and Technology wants genomics, proteomics, and other biomedical researchers to submit ideas about needed advances in personalized medicine, and has asked for white papers detailing these pitches.”

– Genome Web (http://www.genomeweb.com/issues/news/151548-1.html)

#### 2.2.3. Categories and types of consumer personalized medicine

Four areas of consumer personalized medicine are discussed here: direct-to-consumer personalized genomics services, blood and other biomarker testing, environmental testing and predictive biosimulation.

##### Personalized genomics

**Figure 8.**
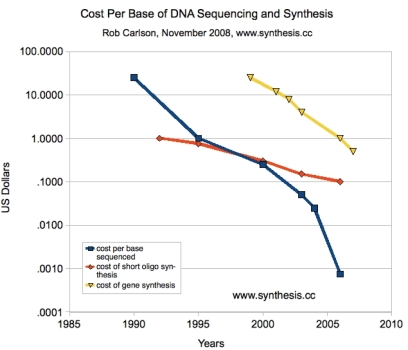
Carlson curves [15].

Personalized genomics is the first and most important area to consider as a key input to consumer personalized medicine. Genomic sequencing came to prominence with the Human Genome Project (http://en.wikipedia.org/wiki/Human_Genome_Project) which ran for thirteen years (1990–2003) and spent $3 billion to sequence the human genome. The human genome has three billion nucleotide base pairs organized into 20,000–25,000 genes. Presently, the two key applications for DNA are sequencing (reading) and synthesizing (writing). Figure 8 shows Carlson curves, the cost per base pair to sequence and synthesize DNA, an analog to Moore’s Law but with faster progress. Advancement could further accelerate as companies like Pacific Biosciences who specialize in next-generation high throughput genomic sequencing technology are estimating that they will have a $100 one-hour whole human genome scan available in 2010 (http://www.pacificbiosciences.com/video_lg.html).

Genomic testing has been available for some time, but was generally ordered by a physician as a one-off test for a particular condition. The shift is in the launch of companies focused on direct-to-consumer genetic testing, offering one-off and multi-SNP array testing of up to one million SNPs, and whole-genome sequencing. A SNP is a single nucleotide polymorphism, an area of known human genetic variation which may be tied to a disease condition. Personalized genomics services are provided by several companies for SNP sequencing including 23andMe (http://www.23andme.com), Navigenics (http://www.navigenics.com), DNA Direct (http://www.dnadirect.com) and deCODEme (http://www.decodeme.com) for prices ranging from $100 to $2,500. At least one company, Knome (http://www.knome.com), affiliated with Dr. George Church’s Personal Genome Project at Harvard University (http://www.personalgenomes.org), provides whole genome testing for approximately $350,000 [16].

**Figure 9.**
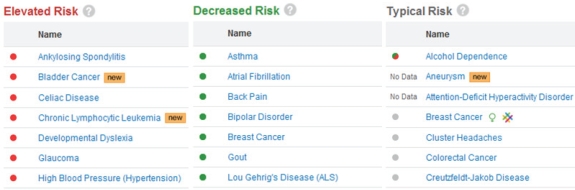
Excerpts from 23andMe’s condition risk profile for a sample patient.

**Figure 10.**
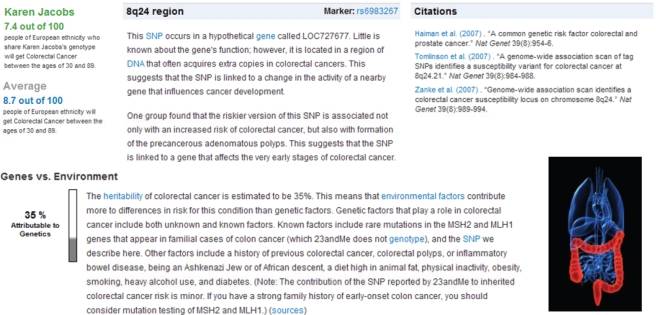
Excerpts from 23andMe’s colorectal cancer marker for a sample patient.

**Figure 11.**
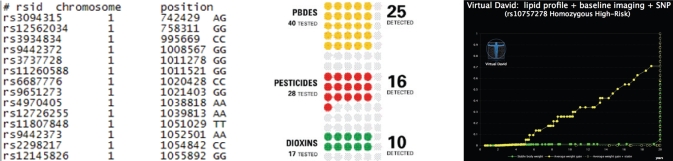
Excerpts of raw genomic data from 23andMe, the environmental scan from the ExperimentalMan project and virtual patient predictive biosimulation output from Entelos.

23andMe is one of the largest and best-known personalized genomics services. From a buccal swab, 580,000 SNPs are scanned and mapped to 78 conditions ranging from heart attack, diabetes and a variety of cancers to lactose intolerance, baldness and earwax consistency. The raw data enumerates the rsID, chromosome number, position and genotype (Figure 11), and is owned and available for download by the consumer. The 23andMe service is ongoing, meaning that after the initial sequencing, there is continued access to the website to see additional conditions and research linked to the genetic data over time. 23andMe and other services could likely offer additional attractively-priced programs to existing members with the advent of whole genome sequencing and other new technologies. So far, several consumers have downloaded their genomic data from 23andMe and other services and posted it to the SNPedia (http://www.snpedia.com/index.php?title=SNPedia), an open source genomics resource which includes the genomes of James Watson, Craig Venter and others. Some views of the 23andMe service are shown in Figures 9 and 10.

Figure 9 displays some of 23andMe’s 78 measured conditions with a sample patient’s indication of higher, lower or normal risk. Figure 10 shows the specifics for the 23andMe colorectal cancer marker. The individual’s risk is shown on a scale of 100 and compared to the average. The specific chromosome region, gene and rsID marker, rs6983267, are given along with citations to current scientific research. Heritable and environmental factors are discussed, with the site estimating that colorectal cancer is 35% attributable to genetics.

In addition to genetic sequencing, there is often a health social networking dimension to personalized genomics communities including the permissioning-in of friends, family and others for information sharing and discussion of genetic specifics. As with other health social networks, clinical trial recruiters are taking advantage of pro-active pre-aggregated populations in personalized genomics communities who have the ability to share genetic marker information. For example, the Michael J. Fox Parkinson’s Foundation approached the 23andMe Parkinson’s community in May 2008 for such studies [17].

As direct-to-consumer personalized genomics is a new field, the longer-term value to individuals is still emerging. Genetic testing is used for disease diagnosis, risk assessment and monitoring, and in evaluating potential drug response, for oneself and in embryo screening, but only works in a limited number of cases. There is often an unclear correlation between genetic information and disease, many diseases are multigenic, having suites of genes potentially involved, and there may be a lack of available therapies if a risk condition does exist. In addition, consumers may risk misinterpreting the information and experiencing false positives, false negatives, or confusion in trying to parse loosely-tied and conflicting research findings. With time, the knowledge and utility of the genome should increase. At present, even the data itself can be a novelty appealing to human curiosity. There is a poignant feeling of individuality when seeing and paging through personal genomic data for the first time, seeing the As, Cs, Gs and Ts that “make you, you.” Also some portion of the information may be useful immediately in the application of maintaining and improving health.

DNA sequencing has also triggered non-health related applications in genealogy, haplotype mapping and immune system-based dating services. In the genealogy segment, companies such as FamilyTreeDNA (http://www.familytreedna.com) and Familybuilder (http://www.familybuilder.com) use DNA testing to provide genealogy-related services. In the dating segment, ScientificMatch (http://www.scientificmatch.com) and GenePartner (http://www.genepartner.com) assess potential mate compatibility based on variation in the immune system’s human leukocyte antigen (HLA) genes, relying on research indicating that stronger pair-bonding and healthier offspring occur in those with dissimilar immune systems [18].

##### Direct-to-consumer blood and other biomarker tests

The second area to measure in consumer personalized medicine is blood and other biomarker data. There is growing consumer demand for much more granular information than the annual check of a few standard biomarkers (blood pressure, cholesterol, etc.) which are compared mainly to peer cohort averages. A small but increasing number of individuals would like to track a wider range of general and personalized indicators on a regular ongoing basis.

In the U.S., there are many online direct access blood test providers offering a variety of services. Two of the largest that have been in business the longest and have the most sophisticated suite of reasonably-priced tests are DirectLabs (http://www.directlabs.com) and the Life Extension Foundation (http://www.lef.org/bloodtest). Both use U.S.-wide LabCorp facilities to perform the tests, the results of which can be viewed online. 43 tests are offered in the basic $97 package from DirectLabs, organized into a complete blood count panel, thyroid panel, lipid panel, liver panel, kidney panel, diabetes panel and other tests. Beyond the general scan, other packages include panels for anemia, arthritis, cancer, cardiovascular health, hormones, immune system and other tests.

There are many benefits to direct access blood testing for the ongoing self-quantifier such as accuracy, low cost, third-party lab professionals and consolidated web data of the current and historical results. At some point it should be possible for individuals to upload LabCorp data to their patient information repositories at health social networks and health portals. As an alternative, there are also many home test kits available for a variety of blood, saliva, urine and other markers, for example testosterone measurement kits (http://www.a1supplements.com/Testosterone-Health-Check-1-Kit-p-17066.html).

##### Environmental testing

The third area to measure in consumer personalized medicine is environmental data, screening for body burden, the cumulative impact of exposure to toxic substances in the environment. Personalized chemical testing services generally focus on pollutants, evaluating the levels of selected pesticides, flame retardants, PCBs, dioxins and other substances. One example is the scan conducted by Axys Analytical Services (http://www.axysanalytical.com) for David Duncan in the ExperimentalMan project (http://www.experimentalman.com/images/chemical_report_card.pdf), an excerpted view of which appears in Figure 11. Environmental screening may also be conducted by hair analysis tests that assess exposure to toxic substances and perform nutritional analysis (http://www.hairanalysisreport.com/hairkit.html).

##### Predictive biosimulation

A fourth area that could be increasingly important to consumer personalized medicine is predictive biosimulation. This is using empirical biological data and mathematical modeling to create an electronic version of an individual, a virtual patient on which to test simulated treatments. Predictive biosimulation is not targeted at consumers yet, other than in the case of a few high-price pilot projects, but could become a valuable personalized medicine technology for individuals. An example of the virtual patient biosimulation is shown in Figure 11, illustrating the baseline case (x-axis) as compared to the expected improvement from a potential intervention (diagonal line). Creating a low-priced or open source version of predictive biosimulation platforms for the self-tracking and software development communities could speed adoption and draw upon the wisdom of crowds to find useful relevant applications as people plug in their various forms of self-obtained data to generate future health scenarios. Two existing predictive biosimulation and virtual patient technology companies are Entelos (http://www.entelos.com) and Optimata (http://www.optimata.com). In December 2008, the U.S. Food and Drug Administration (FDA) announced plans to use the Entelos biosimulation technology to study three heart-related drugs to identify safety and effectiveness issues before the completion of late-stage human trials by testing far more simulated patients than could be included conventionally and obtaining computer-generated test results in days or weeks instead of years [19].

### 2.3. Quantified self-tracking

#### 2.3.1. Quantified self-tracking introduction

**Figure 12.**
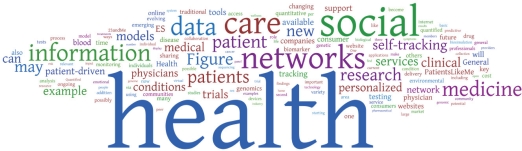
Many Eyes data visualization of this paper.

The increasing ease of capturing, storing and manipulating data has given rise to a variety of websites for sharing datasets and visualization tools, for example IBM’s ManyEyes (http://manyeyes.alphaworks.ibm.com/manyeyes), used here to visualize this paper (Figure 12, word use frequency is indicated by font size), Swivel (http://www.swivel.com) and FlowingData (http://flowingdata.com). In the past, the cost and expertise required for working with large-scale datasets and visualizations generally limited access to institutional professionals, but cost decreases and tool improvements have made data collection and manipulation more available to the individual.

One of the most interesting areas for individuals to measure is the self. At least two interest groups formed in the second half of 2008 to explore, brainstorm and share their self-tracking experiences, the Quantified Self group (http://www.quantifiedself.com) in the San Francisco area and the HomeCamp group (http://homecamp.org.uk) in London. An underlying assumption for many self-trackers is that data is an objective resource that can bring visibility, information and action to a situation quickly, and psychologically there may be an element of empowerment and control. Quantified self-tracking is being applied to a variety of life areas including time management, travel and social communications [20,21], as well as the health context, where the expanded definition of health is embraced as applications address both medical issues and general wellness objectives.

#### 2.3.2. Quantified self-tracking definition

Quantified self-tracking is the regular collection of any data that can be measured about the self such as biological, physical, behavioral or environmental information. Additional aspects may include the graphical display of the data and a feedback loop of introspection and self-experimentation. Health aspects that are not obviously quantitative such as mood can be recorded with qualitative words that can be stored as text or in a tag cloud, mapped to a quantitative scale, or ranked relative to other measures such as yesterday’s rating. Many health self-trackers are recording measurements daily or even more frequently (blood pressure for example).

As with biomarker testing, health metric tracking was traditionally an expensive one-off process ordered by physicians for patients in response to specific medical risks. Two of the biggest applications in doctor-driven health metric tracking are cardiac monitoring and telemedicine (remote diagnosis) where implantable, worn or handheld devices transmit data wirelessly to medical professionals. Contemporary applications were featured at the 2008 USC Body Computing conference (http://www.usc.edu/schools/medicine/departments/medicine/divisions/cardiovascular/bcc) and the Unither Nanomedical and Telemedical Technology Conference (http://www.unithertechnologyconference.com/conferenceagenda08.php). For example, Medtronic has an implantable pacemaker that transmits data using a portable monitor connected to a standard telephone line; physicians check the reading every three months [22]. A constant stream of on-demand pacemaker readings could be useful to both patients and physicians. HealthAnywhere (http://www.igeacare.com/HealthAnywhere/Personal/home.htm) has an implantable monitoring system which provides early warnings of heart failure, transmitting data via cellular telephones and home computers. Mobile health monitoring company MedApps (http://www.medapps.net) is developing Bluetooth broadcasting monitors for common medical devices such as blood glucose meters, pacemakers and scales.

#### 2.3.3. Categories and types of quantified self-tracking tools

Focusing on the consumer self-tracking market, in addition to health social network-based self-tracking, there are a variety of other health monitoring websites and devices currently available. Some of these services and websites are listed in Table 2, they generally have some level of free services but are un-automated, meaning that users must input their own data. The websites may accept data via the Internet, SMS text messaging, instant messaging (IM), smartphone data applications, audio messages or other mechanisms.

The next step in quantified self-tracking health applications is wearable devices with automated data collection (Table 3). There are several devices with similar functionality. The basic feature is accelerometer-detected energy expenditure or calories burned. A second feature provided by some devices is sleep measurement. Most devices are wearable for about a week between battery recharges and require connecting to a computer, usually via USB, to upload the data. The FitBit, estimated to launch in early 2009 will send the data wirelessly to a computer-attached base station.

**Figure 13.**
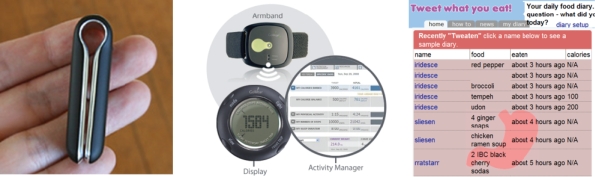
Quantified **s**elf-tracking tools: FitBit, GoWearFit and Tweet what you eat!

There are many other self-measurement devices such as home kits for blood pressure monitoring but data collection is manual. A future can be imagined where it would be standard for all self-measurement devices, for example heart rate monitors, to have an option for wireless network connectivity to automatically download data to a consolidated format for storage, display and analysis. Figure 13 presents visual examples of some of the self-tracking tools discussed above.

#### 2.3.4. Categories and types of environmental tracking tools

**Figure 14.**
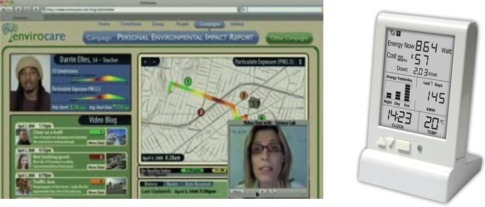
Personal environmental monitoring tools: PEIR and Current Cost.

In addition to tracking biomarkers and behavior, one’s environment is the next logical area to monitor for personal health, resource utilization and other reasons. A side benefit of environmental tracking may be that individuals are encouraged to take responsibility on a larger scale. As with behavioral tracking, there is an interesting array of vendor-provided and consumer-invented tools to facilitate the monitoring process. The first application, environmental monitoring for personal health reasons, takes advantage of consumer mobile phones and sensors connected wirelessly to the Internet. Two key efforts are the UCLA Center for Embedded Networked Sensing’s participatory urban sensing project (http://urban.cens.ucla.edu) which uploads location data from GPS-enabled mobile phones to a central repository and generates Personal Environmental Impact Reports (PEIR) as depicted in Figure 14 with an example of social network members discussing the report. A second effort is OpenSpime’s Internet-connected geosensors, being developed to capture ongoing real-time readings of pollution and other air quality indicators and automatically log the information to a collective display built on Google Maps (http://www.openspime.com).

The second application area, resource utilization, focuses primarily on home automation and utility consumption and management, with projects including electricity, water and solar panel monitoring. Several projects were demonstrated at the November 2008 inaugural HomeCamp (http://homecamp.org.uk), for example the Current Cost in-home electricity monitor (http://www.currentcost.com) pictured in Figure 14, and IBM distinguished engineer Andy Stanford-Clark’s sophisticated home automation system ranging from measuring the electricity consumption to the resistance of cheese in mousetraps, all supported by an extensive messaging and database-driven backend (http://andypiper.wordpress.com/2008/12/01/the-inaugural-homecamp).

## 3. Discussion of Emerging Patient-Driven Health Care Models

One mechanism changing how health and health care are understood and realized is patient-driven health care models, particularly health social networks, consumer personalized medicine and quantified self-tracking. These models support an early shift to patient-driven health care as individuals are starting to measure, track, experiment, intervene, treat and research their conditions and symptoms, genomes, biomarkers, behavior and environment, both individually and in collaboration with others.

This discussion section focuses on several themes and potential future implications that arise from the analysis of emerging patient-driven health care models and includes some speculative and forward-looking thoughts. The areas examined here in more depth are the future of health care and the evolving health care delivery model, the changing role of the patient, the changing role of the physician, the advent of health social networks as influence entities, evolving legal, economic and regulatory institutions, the future of clinical trials via vertical and horizontal stratification and changes in drug discovery and after-market studies.

### 3.1. The Future of Health Care and the Evolving Health Care Delivery Model

The main characteristic of the evolving health care delivery model is that it is starting to become more collaborative; moving to a co-diagnosis, co-care model between physicians, patients and other parties [23]. The physician could start to be seen as a colleague and advisor, as one of many input sources in fashioning a care plan. The patient could become more of an informed participant, an active responsibility-taker, the owner, administrator and coordinator of his or her health program and health data. Future interactions may be those of knowledgeable patients bringing quantitative reports from their self-testing and self-tracking activities to medical professionals for consultative co-interpretation of the results. The concept of health care could be extended to being a complex, ongoing, data-rich process of managing acute, chronic, general wellness and enhancement conditions using a wider variety of traditional and non-traditional health resources such as collaborative peer networks. Health care services could become location and time independent, provider de-linked and commerce-enabled such as American Well’s on-demand web-based physician consultation services with video and chat (http://www.americanwell.com) and the Carol Care Marketplace’s online scheduling and consumer cost management platform (http://www.carol.com) [24].

A second dynamic in the evolving health care delivery model is that large health care institutions and insurance systems may need to change to incorporate the possible coming shift to genomic-based and patient-driven medicine. Physicians are generally not fluent in the new tests and tools of health management such as genomic scans [25], detailed ongoing biomarker analysis, environmental analysis and self-experimentation projects, nor trained to advise on the adjusted concept of health as enhancement rather than cure. There could be substantial reeducation and retraining as institutional medicine adopts the new health care models. New careers in collaborative medicine are already emerging, for example the genetic counselor is one of the fastest growing job categories in any field [26].

A third dynamic in the evolving health care delivery model is managing the information explosion, both for individuals and medical professionals, as the amount and availability of information is increasing. Even specialist physicians may not have time to keep up with every aspect of new research in the fields they cover and certainly general practitioners do not. Some patients have the time and ability to review all available research and information themselves, but there is ample room for other value-chain participants to help consumers navigate and interpret the available information stream. Examples of new value-chain participants that may arise are in-office physician research associates, new categories of medical information providers and expert patients who have developed reputations on health social networks just as community forum leaders and PowerSellers did on other Internet platforms with social dimensions like eBay. Health delivery models are expanding as consumers are using the heightened information flow and interpretation tools to inform their personal health actions.

A fourth dynamic in the evolving health care delivery model is the patient-driven relaxation of privacy. Individuals are not hampered by HIPAA (U.S.-based health privacy legislation), and those that feel comfortable doing so are starting to open source their health information on health social networks. This is creating the significant resource of large public health databases. The privacy convention is opt-in; nothing is compulsory, individuals that would like to share do so. This could lead to more people from different fields looking at health data in new ways, both medically-trained professionals and others, to the possible benefit of all. An example of this is when the Goldcorp company published their proprietary gold mining data on the Internet and had 1,000 virtual prospectors from over 50 countries find 110 targets, 50% of which had not been previously identified by the company and 80% of which yielded new gold reserves [27]. More rigorous large-scale quantitative analysis could possibly inform the discovery and delivery of health care services.

### 3.2. The Evolving Role of the Patient

The role of the patient is starting to shift from being a minimally-informed advice recipient to an active participant, instigating collaborator, information sharer, peer leader and self-tracker engaged in participative medicine; a transition is underway from paternalistic health care to partnership models [28,29]. The small but growing consumer health communities examined in this paper suggest that individuals are becoming more engaged in a variety of self-testing and self-management of conditions, symptoms, genomics and blood biomarkers, behaviors and personal environmental factors. Individuals could dramatically expand their use of web-based tools, devices and health social networking platforms as their awareness increases, costs drop, financial incentives arise and automated tools proliferate. Current and future self-measurement and self-experimentation projects could be formalized into more structured programs, possibly generating even more value in collaboration with others as consumers develop relationships with health social network peers and propose, lead and participate in self-run patient studies.

As data owners and administrators, consumers may be starting to maintain digital files of their medical history either on home computers or by entering the data into available Internet-based electronic medical records (EMRs) to the extent possible given website functionality and personal comfort with online security. The digitized information may include any available documentation from previous doctor visits, a medication history, genomic scans and other biomarker tests, a weight, exercise and sleep history, health journaling information, self-tracking device input and any other relevant information.

Eventually, health social networks, health portals (examples: Google Health, Microsoft HealthVault) or other EMR-specific websites such as Shared Care Plan (http://www.sharedcareplan.org) and Keyose (http://www.keyose.com) could be the application front-ends and centralized repositories of quantitative patient data. These websites could orchestrate EMR access permissions to doctors and other parties. The future might be physicians logging in to patient-owned EMRs to type notes into standardized forms and submit online prescriptions that automatically clear through the patient’s insurance company and automatically fill through the patient’s preferred pharmacy. Even being able to review one’s own medical file online would be a big step. One early version of this is MD VIP (http://www.mdvip.com), who offers a service for patients to input an extended online wellness profile for physicians to review prior to office visits.

Consumers may be evaluating all of their traditional health interactions, for example, identifying the opportunity to approach employers or health insurers for discounts for being healthy, particularly unified as a health social network buying group. Thus far insurance and other aspects of health care have been a one-way street with penalties for pre-existing and negative conditions but no reward or incentive for healthy behavior. Insurance companies have detailed knowledge of patient costs; U.S.-based Humana’s cost to insure people is $11 per pound more per year for each pound overweight they are and $31 per overweight pound per year for obese people 65 and over [30]. Healthy behavior could be demonstrated easily with regular biometric readings such as weight, cholesterol, BMI, blood pressure and other data points. Examples of this idea in implementation include U.S.-based grocery-retailer Safeway who lowered employee health care costs by 13% after instituting a rebate plan for healthy employees or employees who improved their health [31] and the Healthy Incentives program, due to launch in the U.K., which plans to offer a point system to reward BMI reduction and smoking cessation (http://www.launchpad.youngfoundation.org/fund/hia/portfolio/project/healthy-incentives).

### 3.3. The Evolving Role of the Physician

Just as the role of the patient is evolving, so too is that of the physician. The physician may need to adapt to both changing patient behavior and dramatic industry shifts. In interaction with some patient segments, a physician is starting to become a care consultant, co-creator and collaborator, generating health plans together with patients using the new tools [32]. This may involve overcoming the challenge that physicians possibly lack the skills to share health care information and decision-making with patients [33]. A somewhat related Norwegian study found that doctors were most often neutral (55%) as compared to positive (33%) when patients shared peer-generated health content with them [34]. Shared care may have previously connoted greater linkage between health system professionals for coordinated patient treatment [35] but is now also used to mean a direct partnering between physician and patient [36]. Exemplifying the shift to partnership models is the chairman of Radiation Oncology at the Montefiore-Einstein Cancer Center in New York, who tells patients that peer-reviewed information is important and says that “We have to acknowledge that patients do this research. It’s important that instead of fighting against it, we join them and become their coaches in the process [37].” Science, technology and business advancements could change health care so rapidly that many physicians, even those currently in medical school, may not be able to be fully prepared for the shift to genomic-based and patient-driven medicine without becoming actively involved themselves.

Some physicians are starting to understand how health care models could change and seeing ways to offer value to patient-collaborators in the new system. There is a small but increasing number of specialty physicians at private clinics providing a variety of new health services including detailed genomic and blood biomarker analysis and preventive intervention programs, for example Omicia (http://omicia.com, genomics), the Frontier Medical Institute (http://www.fmiclinic.com, longevity), the Los Gatos Longevity Institute (http://www.antiaging.com, longevity) and Amen Clinics (http://www.amenclinics.com, neuro-imaging). In the intervening years before genomics and other biomarker diagnostics and treatments are proven and could be automatically administered via traditional health care channels, they will likely continue to be provided by private clinics as they are now. Consumer demand and the number of clinics could grow, and some sort of services standardization and certification may be appropriate for successful providers to distinguish themselves.

From the growth of medical content in blogs, wikis and other Internet tools such as Twitter (http://www.twitter.com), it is clear that some physicians are participating in defining the broad range of new health care models and in applying Web 2.0 tools in medically relevant ways [38]. A study of junior physicians in the U.K. found that they used Internet tools as a means of being able to find relevant information more efficiently than from other sources [39]. The ScienceRoll Medicine 2.0 blog (http://scienceroll.com/medicine-20) tracks the status and development of some of these innovations. Some recent resources of note have included the Top 50 Health 2.0 blogs (http://acumeme.blogspot.com/2008/12/top-50-health-20-blogs.html), Top Physicians on Twitter (http://www.twitip.com/must-follows-on-twitter-physicians-triathletes-and-horse-people), the Clinical Cases medical blog (http://casesblog.blogspot.com) and a medical Wiki attempting to create a collective online memory for physicians, nurses and medical students (http://www.askdrwiki.com/mediawiki/index.php?title=Physician_Medical_Wiki).

**Figure 15.**
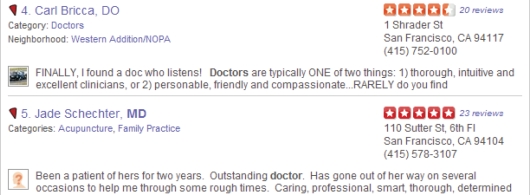
Examples of publicly-available patient reviews of physicians (Yelp).

As some patients are seeing potential health benefits in forgoing their privacy on health social networks, some physicians are also adjusting to a new transparency regime. In the future, doctors could become as concerned about their public reputations as eBay sellers and others depending on online reputations [40]. Review sites for doctors with patient-entered data are proliferating (Figure 15): Yelp (http://www.yelp.com), Angie’s List (http://www.angieslist.com), HealthGrades (http://www.healthgrades.com) and Physician Reports (http://www.physicianreports.com). There may also be reputational aspects to doctor participation in health social network physician Q&A.

### 3.4. The Advent of Health Social Networks as Influence Entities

Peer-based health networks could be poised to become a powerful member of the health care ecosystem with an expanding role, possibly having influence in policy, ethics, regulation, research and finance. It will be interesting to see how health social network identity develops and is expressed since a health social network is simultaneously an aggregation of individuals and an institution with its own leadership, goals and agenda. In other sectors, social networks have sought to maintain neutrality by “only providing the platform,” for example peer-to-peer finance sites like Prosper (http://www.prosper.com). It is too early to forecast what will happen with health social networks, but PatientsLikeMe as the flagship example has an on-site research staff and appears to be quite involved in administering and orchestrating the patient community, with a collaborative stance towards traditional medicine. Internet-expert Clay Shirky notes the progressive stages of social network activity which seem to be unfolding in lockstep in health social networks: initially sharing, then collaborating, and finally organizing for collective production and collective action (http://www.herecomeseverybody.org). In addition to external collective action, the internal peer support of health social networks could evolve into positive-impact peer pressure, for example, members competing to lower key biomarker scores like cholesterol and blood pressure, using third-party test uploads from LabCorp to measure and validate the results.

Health social networks could develop into large-scale online aggregated communities with market power, providing visibility into demanded research and remedies and directing and funding research priorities. One future example could be the CureTogether migraine community raising $50,000 in crowd-sourced funding, reviewing and approving grant applications, open-sourcing the research findings on their website and developing and testing remedies in patient-run clinical trials. Health social networks could become a key quantitative indicator and independent barometer of demand for medical research, a useful input to the research agenda-setting of governmental funding bodies such as the National Science Foundation (NSF) in the U.S., pharmaceutical companies and academia.

One of the biggest potential benefits of health social networks could be in making long-tail medicine possible, allowing small communities to find each other via the Internet. The long-tail concept (http://en.wikipedia.org/wiki/The_Long_Tail) is that with traditional models, it was only economically feasible for service providers to address the largest 20% of the market. Internet models allow the other 80% of the supply and demand curves to come together too. For example, Amazon and Netflix, providing books and movies by mail, are able to stock 100% of possible items in their inventories whereas the cost constraints of brick-and-mortar booksellers and movie rental companies only allow them to stock the top-selling 20% of items. The cost structure of large pharmaceutical companies is just like that of brick-and-mortar stores and record companies; they need blockbuster products with significant sales. Peer-to-peer health social network models could open this up substantially and address orphan diseases and what may be the other 80% of the market. Individual researchers, small to mid-size pharmaceutical companies and foundations could start to quantify demand for non-marquee health conditions and work on solutions collaboratively with health social network patient communities, possibly also funded by the patient communities.

There are several ways that health social networks could exercise their power as market agents. One idea is for health social network members to come together in aggregated buying groups for discounts and Request for Proposal (RFP) solicitations from vendors for health insurance, medical services, gym memberships, vitamin supplements and other health-related purchases. Health social networks could serve as the platform for virtual services such as on-demand physician consultation. Another idea is for health social networks, in addition to their participation in regular clinical trials, to become involved with the ongoing testing of non-drug remedies such as supplements, vitamins, minerals, nutraceuticals, herbal remedies and other products; testing both contents claims and efficacy. This could work both ways; supplement companies could contract with health social networks for studies to discover and demonstrate the benefits of their products and health social networks could periodically review all large supplement providers, like Consumer Reports (http://www.consumerreports.org) with more rigorous health-benefit testing. Health social networks could provide a variety of independent product certifications such as “Remedy X is MedHelp tested and approved,” or “3 out of 4 MedHelp patients say they use Remedy Y and have experienced these results...” Health social networks could also be a useful platform for multi-factor longitudinal studies where it may take years to assess the impact of lifestyles, interventions and behaviors such as taking certain groups of vitamins. Studies would not need to be designed at the outset but could be derived from on-demand database queries to probe for connected factors and groups of patients with similar profiles.

Translational medicine, converting basic research findings to patient therapies, continues to be one of the greatest outstanding problems in medicine and health social networks might be able to be helpful here too. One way is by connecting researchers with health social networks such that there is more of a direct and continuous feedback loop between the needed research and the executed research. A key step may be missing from the current translational medicine process that expert patients and health social network researchers could help with, the enumeration of the specific functionalities or capabilities of the research findings in a context such that non-scientists who are knowledgeable in the area can more easily see what solutions those capabilities could generate, the “productizing” of the research findings. There could be an opportunity to connect researchers, medical professionals, patients, health social network representatives, industry analysts and other parties in Translational Medicine Advisory Boards, akin to the Technology Advisory Boards used by basic research institutions in the technology industry, to facilitate translational medicine.

Consumers are uniquely positioned to tackle some other key health care industry issues. Just as patients are the only ones who can avoid HIPAA privacy regulations and open source their own data to the benefit of the greater community, patients can skirt the social taboos that other health care ecosystem members may encounter regarding economic issues. Patient-driven health social networks could promulgate cost rationalization by demanding price lists from health service providers and backing consumer credit programs for health care debt such as those envisioned by Criterion Ventures [41]. Much of preventive medicine may continue to be non-reimbursed; one positive aspect of this is that providers would be forced to develop consumer-presentable health service offerings and pricing.

Finally and on a more speculative note is one of the other major advancing fields of life sciences, synthetic biology, also known as bioengineering (http://openwetware.org). Essentially, synthetic biology is printing synthesized sequences of DNA after designing them by computer, possibly using the open source biological parts database (http://partsregistry.org). This could be relevant to the future of patient-driven health care if individuals or health social networks were to promote the do-it-yourself home synthesis of test substances, for example printing measurement chemicals for blood and saliva tests used in the self-monitoring process.

### 3.5. Changing Legal, Economic and Regulatory Institutions

Legally, non-discrimination laws such as the Genetic Information Nondiscrimination Act (GINA) passed in May 2008 in the U.S. (http://www.genome.gov/24519851) exist but many people may still be worried about potential genetic discrimination from employers and insurance companies. Given the rate of advancement in DNA sequencing technology, it might be possible that whole genome scans become as normal as blood tests in the health system before laws like GINA are tested and a legal precedent is set regarding genetic discrimination.

Economically, a trend is underway for employers to switch from traditional health plans to health savings accounts (HSAs) (http://en.wikipedia.org/wiki/Health_savings_account) as a means of lowering their health care costs. More than half of U.S. large-company plans will offer an HSA as an insurance option in 2008–2009 [42]. HSAs make end-users of health care services more aware of pricing and there is an ongoing debate about whether they produce better or worse health outcomes, but it is possible that consumers would be more efficient in their consumption of services behaving directly as rational economic agents. To the extent possible, consumers may choose to put their HSA dollars to work in new ways, focusing on preventive medicine and other emerging patient-driven health care solutions. Employers could benefit by implementing HSAs together with employee health rebate incentives.

Regulatory controversy, the struggle between the old medicine and the new medicine, has already been part of the emerging patient-driven health care landscape in that many direct-to-consumer products and services are unregulated. Opined as initiated by the traditional medical establishment, in June 2008, California served cease and desist orders to direct-to-consumer personalized genomics companies [43]. As of early July 2008, apparently only five of thirteen such companies in California had complied with the order, arguing that they were not in infraction of any laws [44] and in August 2008, two companies were licensed in California as genetic information services providers, as distinct from medical testing providers, 23andMe and Navigenics [45]. It will be interesting to see whether the traditional medical industry strikes a symbiotic or adversarial stance as additional patient-driven health care models emerge.

Regulation could continue to be a bottleneck. Agencies like the U.S. Food and Drug Administration (FDA) and the European Medicines Agency (EMEA) are already taxed in responding to the high number of clinical trials and new drug approval requests; in December 2008, the U.S. National Institutes of Health Clinical Trials website listed 65,241 clinical trials in process (http://clinicaltrials.gov) as compared to 48,826 at the start of the year (http://web.archive.org/web/20080102061002/http://clinicaltrials.gov). There could be even more of an effort to organize the process into different tiers with fewer requirements depending on the therapy type. New approval models with peer-based research and support components, analogous to those proposed for patents by Beth Novak (http://www.peertopatent.org) and Lawrence Lessig (http://remix.lessig.org) could be helpful in easing regulatory burdens.

### 3.6. The Future of Clinical Trials: Vertical and Horizontal Stratification

Clinical trials are the biggest cost in the estimated $1.3 billion total cost of bringing each new drug to market [46]. Personalized medicine could trigger the need for more levels and types of cohort testing in both regulatory agency-approved clinical trials and open source patient-run clinical trials. It may make sense to have a wider array of vertical test tiers to accommodate more alternatives to the full-blown clinical trial such as informed patient field studies, small-group phased experiments and data collection projects. Horizontally, there may need to be as many as exponentially more cohort studies to segment varying genetic, phenotypic biomarker and patient history data as the early drugs of personalized medicine, Imatinib and Tamoxifen, are starting to show.

The clinical trials process could be expedited dramatically with patient-driven medicine. Health social networks can bring pre-aggregated patient registries and standardized digital data to clinical trial conductors. Health social networks and clinical trial representatives can collaborate in their needs for electronic data collection, ensuring that the quantitative information needed for clinical trials already exists for all participating patients. Clinical trial pre-screening surveys can be administered easily through health social network websites. Related to the quantitative shift in medicine, future drug approvals may require biosimulation modeling and analysis output in addition to live patient data as a standard application component; the use of biosimulation by regulators is already being explored as discussed in Section 2.2.3. Predictive biosimulation.

### 3.7. Changing Drug Discovery and After-Market Studies

The quantitative data repositories of health social networks could become a significant resource in the drug discovery process too as researchers start to be able to cross-reference large databases of genomes, biomarker readings and patient histories. The ongoing self-tracking activities of health social network members might fit nicely with the needs of after-market drug studies. Patients are already logging their experience, adherence and side effects which could then be aggregated to be visible publicly on the Internet instead of collected in a more costly way and possibly presented in a legal but potentially biased way by pharmaceutical companies.

In the future, personalized drug dosage could come to mean custom tuned for each individual per their own biomarker readings at the time of the dosing. Smart dosing nanomedicine drug delivery systems could dynamically update dosage profiles over the life of the drug. What starts as a whole genome scan of each person could eventually lead to individuals having full electronic health models of themselves, updated automatically in near real-time per their biomarker readings. On-demand treatment biosimulations could be run with virtual patient models from Entelos, Optimata or others. Individuals could open, review and adjust their 3D health models in virtual worlds such as Second Life (http://www.secondlife.com), for example modeling current nutrition and exercise profiles over time from actual self-tracking data and zooming in to walk through their own arteries to observe first hand atherosclerotic build up and other aspects. A current example of such 3D models of the body is the virtual testis exhibit in Second Life (http://www.youtube.com/watch?v=O1YuRSyzBAE) where biological processes can be experienced from the viewpoint of being inside the body.

### 3.8. Discussion Summary of Emerging Patient-Driven Health Care Models

In summary, the key points from this discussion of the themes and potential future implications of emerging patient-driven health care models are that a collaborative co-care model is starting to evolve for health care delivery and that the patient’s role may become one of active participant, information sharer, peer leader and self-tracker, while the physician’s role may become one of care consultant, co-creator and health collaborator. All parties may face a sea change of adaptation in the coming era of genomic-based medicine. One of the most important dynamics in the transition to patient-driven health care could be the advent of health social networks as influence entities as they may increasingly conduct self-run clinical trials, wield market power through aggregated buying groups, serve as an indicator of market demand for medical research and possibly lead translational medicine. Legal, economic and regulatory institutions too may need to innovate to manage the shift to genomic medicine and patient-driven health models. The future of clinical trials could mean much greater vertical and horizontal stratification with exponentially many cohorts. Clinical trials, drug discovery and after-market studies could be expedited by the growing public health databases of patient-contributed information.

## 4. Conclusions

The introduction posited that the life sciences field is changing in several important ways both content-wise and structurally. Emerging patient-driven health care models are influencing some of these changes and could contribute to shaping a positive future for life sciences and health care. Content-wise, one key life sciences change is the growth in information, both in the amount of general and scientific information, and in the type of information, as narrow and interdisciplinary scientific fields expand. Patient-generated content in emerging health care models is adding a new category and dimension of information. Consumers are not just creating information but also helping to make it meaningful and navigable, first by organizing themselves and the information into knowledge communities. Second, at some websites, individuals creating or interacting with the data can help to stratify it with relevancy and abstraction layers by actively engaging in collaborative filtering, tagging, voting and other standard Internet community data management techniques or passively, by having their attention recorded as page views. Third, individuals such as expert patients are becoming value-added health information resources themselves through their self-knowledge and community participation. Health social networks and other peer-based resources such as patient registries by condition could proliferate and be formalized into tools analogous to the Wikipedia which has emerged as a widely useful consumer-produced information resource.

Structurally, the life sciences field is changing in three ways, the concept of health, how science is being conducted, and the models by which health care is realized. In the first case, the notion of health is changing as patient-driven models help to expand the definition of health and health care as depicted in Figure 1. Individuals may have the time, interest and utility to tinker with different tiers of the health concept, thinking creatively about and experimenting with cures, especially for pharmaceutically-uninteresting or complex orphan diseases, improving and resolving chronic conditions, measuring and reaching baseline normalization, increasing wellness, preventing disease and engaging in genetic and physical enhancement as possible.

In the second case, how science is being conducted and who is conducting it is also being influenced by patient-driven health care models. Individuals can focus on personally-relevant aspects of health, formulating important and possibly novel areas of inquiry, collecting data about their experiences and finding others with similar interests and conditions with whom to collaborate and mobilize resources. Rather than forming a hypothesis at the outset, individuals may engage in self-tracking, analyzing the resulting data and using self-experimentation as a tool for improvement; for the individual, understanding the underlying mechanism may be irrelevant if desired outcomes are obtained. Mathematical modeling, simulation and synthetic biology are also redefining and adding to the way that science is being conducted by traditional professionals and could potentially have an even more powerful impact if they were to be made available in easy-to-use consumer offerings. Many tools are freely available but not packaged in accessible ways for different user groups, for example Stanford’s SimTK biological structure simulation models (http://www.simtk.org) and synthetic biology’s DNA parts database which contained over 3,500 standardized building blocks as of December 2008 (http://www.partsregistry.org).

In the third case, the ways that health care is realized are also changing through patient-driven health care models. Early examples include health social networks, direct-to-consumer personalized medicine services, self-tracking communities and non-reimbursed clinics for preventive medicine and other interventions. The creative exploration of individuals focusing on a much wider concept of health could further add to the value chain of health services, extending them from the traditional model of general diagnostic and urgent care providers with high expertise (e.g.; physicians and hospitals) to new areas. An interesting array of alternative models and entrepreneurial services could arise, for example on-demand physician consultation as a standard service, personalized genomics interpretation and intervention offerings as genetic information becomes more clinically relevant, and fully automated tools for personal quantified self-tracking and environmental monitoring.

### 4.1. Summary and Opportunity

The growing presence of patient-driven health care models may be central to the evolving health ecosystem. Individuals are starting to better manage their health, independently, with peers, in large aggregated online affinity communities and in consultative co-care with medical professionals. Tools, demographics and financial incentives may combine to accelerate the achievement of improved health outcomes for all ages. Individuals and groups of individuals as new classes of participants in the health ecosystem could be beneficial at many levels from the practical, inspiring the launch of resources, services and businesses, to the theoretical, helping to inform the general inquiry of health and to supplement the traditional scientific method with empirical data.

More health resources and alternatives are starting to be available, consumers can control more of their own data and are becoming empowered to make their own choices; traditional medicine is no longer the exclusive source of health solutions. The individual can obtain relevant information more readily and act upon it. Health information databases and patient registries by condition are emerging as a significant public resource.

The emerging patient-driven technology-enabled health care models have focal points at every node of the wellness cycle, particularly at earlier stages, targeting prevention rather than therapy. Uptake could advance quickly given the more open attitudes of younger generations regarding trust and privacy and their facility in using Internet models for information-seeking, communication and action-taking. Self-collected digital data could be an input to quantitative analysis, predictive outcomes and biosimulation. Consolidated reflection on reductionist self-measurement activities could be extrapolated into new perspectives such as a shift in the overall conceptualization of health, and the meaning of wellness to the individual and society.

For both consumers and all manner of medical and public health and environmental research professionals, this could be a time of great opportunity. There is a potential chance to learn and apply the emerging models, to invent new tools, to reach out to a global peer audience in collaboration, to embrace technological change and to make progress on systemic challenges that may have previously appeared intractable.

## Figures and Tables

**Table 1.** List of health social networks (information current as of 12/23/08).

Legend:	QS: quantified self-tracking
ES&IS: emotional support and information sharing	CT: clinical trials access
Q&A: physician Q&A	N&I: networking and information

**Name and URL**	**Focus**	**Services**
**General health social networks**		
PatientsLikeMe http://www.patientslikeme.com	General: 16 conditions, ex: ALS, MS, HIV/AIDS	ES&IS, QS, CT
CureTogether http://www.curetogether.com	General: 175 conditions, ex: endometriosis, migraine	ES&IS, QS
MedHelp http://www.medhelp.org	General: tracking, physician Q&A	ES&IS, Q&A, QS
Inspire http://www.inspire.com	General: over 1,000 health and wellness groups, journaling	ES&IS, CT
DailyStrength http://www.dailystrength.org	General: 600+ conditions	ES&IS
OrganizedWisdom http://www.organizedwisdom.com	General: 60 conditions, referenced research citations	ES&IS
WellSphere http://www.wellsphere.com	General: 50+ communities, ask an MD feature	ES&IS, Q&A
MDJunction http://www.mdjunction.com	General: 750+conditions, physician listings and marketplace	ES&IS, Q&A
ehealth forum http://ehealthforum.com	General: physician Q&A; 1.2M questions logged on the site	ES&IS, Q&A
HealthChapter http://www.healthchapter.com	General: 715 groups	ES&IS
Trusera http://www.trusera.com	General: 23 main topic areas	ES&IS
iMedix http://www.imedix.com	General: information, support and answer response	ES&IS, Q&A
Wellescent http://www.wellescent.com	General	ES&IS
WeGoHealth http://www.wegohealth.com	General: answer response from health activists	ES&IS, Q&A
Experience Project http://www.experienceproject.com	General: members, stories, discussions	ES&IS
Peoplejam http://www.peoplejam.com	General	ES&IS
**Cause-specific health social networks**		
TuDiabetes, http://tudiabetes.com dLife,http://www.dlife.com SugarStats, http://www.sugarstats.com	Diabetes; SugarStats offers self-tracking	ES&IS ES&IS ES&IS, QS
Prostate Cancer Info Link http://prostatecancerinfolink.ning.com	Prostate cancer	ES&IS
I’m Too Young for This http://www.imtooyoungforthis.org	Youth with cancer	ES&IS
CareFlash, http://www.careflash.com Care Pages, http://www.carepages.com	Personal webpages to share health updates with family and friends	ES&IS
WeAreDiabetic.org, WeAreCeliac.org http://weare.us	General/support: 14 communities	ES&IS
**Physician social networks**		
Sermo http://www.sermo.com	General: free membership to verified physicians	N&I
Medscape Physician Connect http://www.medscape.com http://tiny.cc/pRhuj (detail)	General: free membership to verified physicians	N&I
Asklepios http://www.asklepios.ca	General: Canadian physician networking and collaboration tool	N&I
MedicalExchange MEDTING http://www.medting.com	Online research, publishing and case collaboration with images and videos	N&I, Collaboration
OR-Live http://www.or-live.com	Online surgical and health care video and webcast communication platform	N&I, Collaboration
The Doctor’s Channel http://www.thedoctorschannel.com	Doctors learning from each other via short streaming video clips	Education
Additional medical industry physician and scientist social networks: http://scienceroll.com/2008/05/24/community-sites-for-scientists-and-physicians-the-list		

**Table 2.** Health-related self-tracking websites and services.

**Service**	**Function**
Trixie Tracker http://www.trixietracker.com	Track and display the activity patterns of babies; sleeping, eating, diaper changes, etc. (website)
MyMonthlyCycles http://mymonthlycycles.com Mon.thly http://mon.thly.info Ovulation.com http://www.ovulation.com	Track menstrual cycles, use fertility calendars (websites and smartphone (iPhone, G1) applications)
BedPost http://www.bedposted.com	Measure sex frequency, duration, quality (website)
Tweet what you eat! http://www.tweetwhatyoueat.com	Maintain a food diary with updates through SMS text messages, instant messages or web input. Calorie count suggestions come from a crowd-sourced database (website)
Zume Life’s Zuri http://www.zumelife.com	Generate medical reminders and track medication, biometrics, food, exercise and health status. Customized health tracking via a speech transcription service (website, device and iPhone application)
Keas http://www.keas.com	Highly personalized full health and wellness profiles, ongoing lab results, custom questionnaires and interaction with physicians and other health providers (website, due to launch in 2009)

**Table 3.** Wearable self-tracking devices with automated data collection.

**Product**	**Functions**	**Connectivity**	**Pricing**	**Availability**
FitBit http://www.fitbit.com 2 inch belt unit	Energy expenditure, sleep measurement	Wireless	$99 one-time fee including device + ongoing website access	Available Q1 2009
GoWearFit http://www.gowearfit.com Arm band + display watch	Energy expenditure, sleep measurement	USB	$159.95 + $6.95/month website subscription fee	Available now
Bodybugg http://www.bodybugg.com Arm band + display watch	Energy expenditure	USB	$249 + $14.95/month website subscription fee	Available now
WalkingSpree http://www.walkingspree.com Pedometer	Energy expenditure	USB	$99.95	Available now
ActiHealth http://www.actihealth.com	Blood pressure, weight, activity	USB, Bluetooth	$130 pedometer	Not yet available
Tanita http://www.tanita.com/radiowirelessscales.shtml	Wireless bathroom scales	Bluetooth	$249.99	Available now
OrSense http://www.orsense.com	Non-invasive spot and continuous glucose monitoring	Manual	N/A	Not yet available
Kickbee http://portfolio.menscher.com/itp/kickbee	Tracking of baby kicks from the womb	Sensor, Bluetooth	N/A	Not available commercially
